# In Vivo Detection of PARACEST Agents With Relaxation Correction

**DOI:** 10.1002/mrm.22340

**Published:** 2010-04-23

**Authors:** Craig K Jones, Alex X Li, Mojmír Suchý, Robert H E Hudson, Ravi S Menon, Robert Bartha

**Affiliations:** 1 Centre for Functional and Metabolic Mapping, Robarts Research Institute, The University of Western OntarioLondon, Ontario, Canada; 2 Department of Medical Biophysics, The University of Western OntarioLondon, Ontario, Canada; 3 Department of Chemistry, The University of Western OntarioLondon, Ontario, Canada

**Keywords:** PARACEST, in-vivo, kidney, OPARACHEE, contrast agent, MRI, chemical exchange, relaxation, mouse

## Abstract

Several pulse sequences have been used to detect paramagnetic chemical exchange saturation transfer (PARACEST) contrast agents in animals to quantify the uptake over time following a bolus injection. The observed signal change is a combination of relaxation effects and PARACEST contrast. The purpose of the current study was to isolate the PARACEST effect from the changes in bulk water relaxation induced by the PARACEST agent in vivo for the fast low-angle shot pulse sequence. A fast low-angle shot–based pulse sequence was used to acquire continuous images on a 9.4-T MRI of phantoms and the kidneys of mice following PARACEST agent (Tm^3+^-DOTAM-Gly-Lys) injection. A WALTZ-16 pulse was applied before every second image to generate on-resonance paramagnetic chemical exchange effects. Signal intensity changes of up to 50% were observed in the mouse kidney in the control images (without a WALTZ-16 preparation pulse) due to altered bulk water relaxation induced by the PARACEST agent. Despite these changes, a clear on-resonance paramagnetic chemical exchange effect of 4-7% was also observed. A four-pool exchange model was used to describe image signal intensity. This study demonstrates that in vivo on-resonance paramagnetic chemical exchange effect contrast can be isolated from tissue relaxation time constant changes induced by a PARACEST agent that dominate the signal change. Magn Reson Med 63:1184–1192, 2010. © 2010 Wiley-Liss, Inc.

Chemical exchange saturation transfer (1) can be used to detect endogenous protons (e.g., amide protons (2)), as well as amide or water protons bound to exogenous diamagnetic (3) and paramagnetic (4) agents that exchange with bulk water protons. Paramagnetic chemical exchange saturation transfer (PARACEST) agents have been designed to be sensitive to physiologic parameters such as pH and temperature (1, 5–8), metabolites of interest (4, 9), or proteins such as fibrin (10). In all cases, exchangeable protons are not directly detected. Rather, they are selectively saturated by the application of a long radiofrequency pulse applied at the resonance frequency (δω) (11) of the exchangeable proton, provided the slow exchange condition (δω · τ <1, where τ is the lifetime of the protons bound to the PARACEST agent) is satisfied (1). These saturated protons exchange with bulk water protons, decreasing the detectable bulk water signal. More recently, an alternative approach was introduced (12) to detect PARACEST agents in the presence of faster exchange. This method involves the on-resonance excitation of bulk water such that spins that do not exchange to off-resonance sites are rotated by a multiple of 360°. Spins that exchange to off-resonance sites do not experience the complete frequency-selective radiofrequency pulse and are incompletely refocused, thereby reducing the bulk water signal. This method has been termed on-resonance paramagnetic chemical exchange effects (OPARACHEE) and is directly related to the agent concentration (12). The OPARACHEE mechanism has several advantages over off-resonance PARACEST detection, including sensitivity to all bound protons that are exchanging with the bulk water and reduced power requirements (12). The main disadvantage is that sensitivity to physiologic parameters based on the chemical shift or shape of the bound proton resonance is lost.

The detection sensitivity of chemical exchange saturation transfer agents depends on many factors, including the chemical shift of the exchangeable proton, the rate of exchange, the saturation power level, and the duration of the saturation pulse (1, 13). PARACEST agents offer the greatest potential sensitivity due to the large chemical shift of the exchangeable protons, which allows greater exchange rates while satisfying the slow exchange condition (δω · τ <1) for off-resonance detection. Previous studies have estimated the detection sensitivity of PARACECT agents by the OPARACHEE mechanism (12) or off-resonance saturation (13) to be in the micromolar range. However, PARACEST agents with exchangeable proton chemical shifts <150 parts per million (ppm) have significantly reduced sensitivity in vivo due to the concomitant magnetization transfer effects from endogenous macromolecules (15), particularly at the high power levels required for off-resonance saturation or OPARACHEE preparation. Despite the reduced sensitivity, several studies have reported in vivo detection of PARACEST agents (16, 17) following high-dose (100 μL of 20-50 mM) intravenous injection. These studies identify image signal intensity (SI) changes over time in kidney and liver following injection of thulium-based agents using the OPARACHEE contrast mechanism. In one case, a spin-echo pulse sequence with a pulse repetition time (TR) of 1 sec (17) was used to detect signal change as a function of time; however, faster imaging schemes based on the true fast imaging with steady state precession (TFISP) pulse sequence have also been used (16).

Although previous studies have demonstrated PARACEST contrast agent detection, the observed signal changes included the effects of altered bulk water *T*_1_ and *T*_2_ in addition to the PARACEST effect. Signal changes resulting from decreased *T*_1_, *T*_2_, or 

 (1) relaxation time constants may enhance agent detection in dynamic studies. Failure to account for these relaxation-based signal changes diminishes one of the primary advantages of PARACEST agents: the ability to turn the contrast on and off at will (1, 13). Therefore, the purpose of the current study was to characterize the relaxation induced signal changes for thulium-based OPARACHEE contrast agents, to demonstrate that the OPARACHEE effect can be isolated in vivo in the mouse kidney, and to create a model of the PARACEST and relaxation effects that fully describe SI variations in time-course studies acquired using a fast low-angle shot (FLASH) pulse sequence. The FLASH pulse sequence was chosen for this study as it can be used to acquire images rapidly, with minimal artifacts.

## MATERIALS AND METHODS

### Bovine Serum Albumin Phantoms

A PARACEST agent containing a Tm^3+^ cation, cyclen ring, CH2C(O) linker, and the Gly-Lys dipeptide sequence as functionalized side chains (Tm^3+^-DOTAM-Gly-Lys) was synthesized as described previously (18). Phantoms containing 0-, 2-, 6-, and 10-mM Tm^3+^-DOTAM-Gly-Lys (18) in phosphate buffer solution and 10% cross-linked bovine serum albumin (pH = 7) were created in NMR tubes. *T*_1_ and *T*_2_ relaxation time constants were measured on a 9.4-T vertical-bore Varian (Varian Inc., Palo Alto, CA) NMR spectrometer at 37°C using an inversion recovery pulse sequence (inversion time = 0.0125, 0.025, 0.05, 0.1, 0.2, 0.4, 0.8, 1.6, 3.2, 6.4, 12.8, 25.6 sec; TR = 20 sec) and a multiecho spin-echo sequence (echo time = 0.00125, 0.0025, 0.005, 0.01, 0.02, 0.04, 0.08, 0.16, 0.32, 0.64, 1.28, 2.56 sec; TR = 20 sec), respectively. The *T*_1_ and *T*_2_ relaxation time constants were quantified by fitting the mean SI in each phantom to the standard relaxation curve using the fmincon optimization algorithm in Matlab (The MathWorks, Natick, MA). The PARACEST effect was isolated from relaxation induced signal changes by taking the difference in the NMR SI between an acquisition preceded by a 240-ms, 6-μT WALTZ-16 preparation pulse and a second acquisition without a preparation pulse. The effect of PARACEST agent concentration (OPARACHEE effect) on the mean NMR SI was quantified using an exponential model.

The phantoms described above were imaged using a multiecho (eight echoes) gradient echo pulse sequence (TR = 14.9 ms, echo time = 1.4, 3.1, 4.7, 6.4, 8.0, 9.7, 11.3, 13.0 ms) on a horizontal-bore 9.4-T Varian MRI scanner. The proton density (PD) and 

 time constant were calculated for each phantom by fitting to the signal equation:(20)


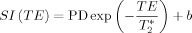
(1)

where SI is the average image SI corresponding to the specified echo-time, *b* is the baseline offset, and the other parameters are described above. The fmincon routine in Matlab was used to minimize the sum of squared errors.

### Mouse Kidney Time Course

To measure the in vivo OPARACHEE effect, 16-g Balb/C mice (*N* = 4) were imaged using a 9.4-T Varian Inova MRI scanner. This study was approved by the University Council of Animal Care at the University of Western Ontario. Each mouse was placed in an anesthetic induction chamber and anesthetized using 4% isoflurane in oxygen. The mouse was then transferred onto a nose cone and maintained on isoflurane at a rate of 1.5-2.5%, oxygen 2 L/min. A catheter fed by two separate lines was placed in the tail vein. The first line contained saline, while the second line contained contrast agent in saline adjusted to pH 7. The mouse was placed prone in a 30mm millipede coil (Varian) and the abdomen of the mouse was lightly taped to the coil to minimize movement and breathing artifacts. Throughout imaging, the mouse's respiration rate and body temperature were monitored using an MR-compatible physiologic monitoring and gating system (SA Instruments Inc., Stony Brook, NY). With the use of a warm-air feedback system, animals were maintained at 37.5°C. Single-slice FLASH images (TR/echo time = 4.7/2 ms, 128 × 128 centric readout, 30mm field of view, two-dimensional single slice 1mm thick) were acquired continuously for 70 min through the left and right kidneys. Every second image was preceded by a 240-ms, 6-μT WALTZ-16 pulse (19) to generate OPARACHEE contrast. An interimage delay time of 3 sec allowed time for *T*_1_ relaxation. Following the first 5 min of continuous imaging, 150 μL of 50-mM Tm^3+^-DOTAM-Gly-Lys (*N* = 2) or 50 μL of 50-mM Gadolinium diethylenetriamine pentaacetic acid (Gd-DTPA) (*N* = 1) was injected.

Images were two-dimensional motion corrected using an in-house registration algorithm. The superior edge of the kidney was tracked throughout the time series and the position was shifted in the superior-inferior direction so that the superior edge was at the same voxel location. Regions of interest were manually selected in the medulla and cortex of the kidneys, and the average SI was measured as a function of time after normalization to the mean SI at baseline (0-5 min). SI curves were generated separately for the FLASH images (*SI*_*FLASH*_(t)) and for the FLASH images preceded by the WALTZ-16 saturation pulse (*SI*_*WALTZ*-*FLASH*_(t)). OPARACHEE contrast was calculated at each time point using the expression (*SI*_*FLASH*_ (t) − *SI*_*WALTZ*-*FLASH*_(t))/*SI*_*FLASH*_ (t) × 100 and then subtracting the mean baseline signal (t <1 min).

The concentration of PARACEST agent observed in vivo was calculated using an exponential relation between OPARACHEE percentage change and concentration calculated for the Tm^3+^-DOTAM-Gly-Lys phantoms described above. This exponential relation was applied to the OPARACHEE contrast observed in the mouse kidney to quantify the concentration of PARACEST agent throughout the time course.

### Mouse Kidney Relaxation Measurement

To further understand image SI changes generated by the PARACEST agent, tissue 

 time constants and proton densities were measured following agent injection in a fourth 16-g Balb/C mouse. Following animal preparation as described above, a two-dimensional multiecho (eight echoes) gradient echo pulse sequence (TR = 14.9 ms, echo time = 1.4, 3.1, 4.7, 6.4, 8.0, 9.7, 11.3, 13.0 ms) was used to image the kidneys and bladder. All other imaging parameters were as described above. The multiecho acquisition was repeated 900 times (total acquisition time was 70 min); every second acquisition was preceded by a 240-ms, 6-μT WALTZ-16 preparation pulse. The PD and 

 were quantified by fitting to the signal equation (Eq. 1). The calculated 

 and PD time courses were median filtered with a kernel width of 5 to remove spike noise.

### Modeling the SI Time Course

The SI time course of the FLASH-based OPARACHEE contrast following agent injection was modeled using the Bloch equations incorporating the four-pool exchange terms (Table 1) (15) and relaxation parameters based on the FLASH signal equation. Two SI time courses, 
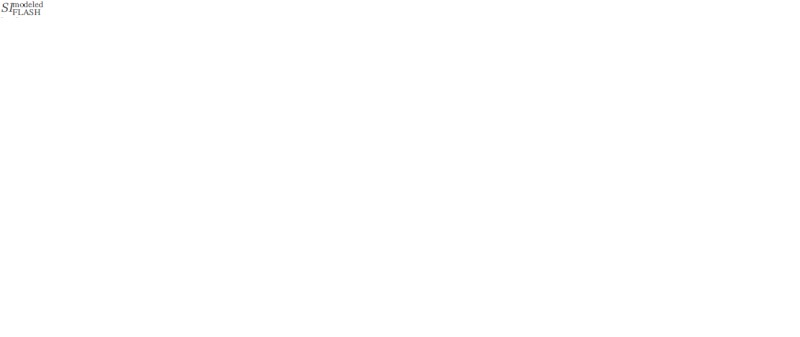
 (*t*) and 
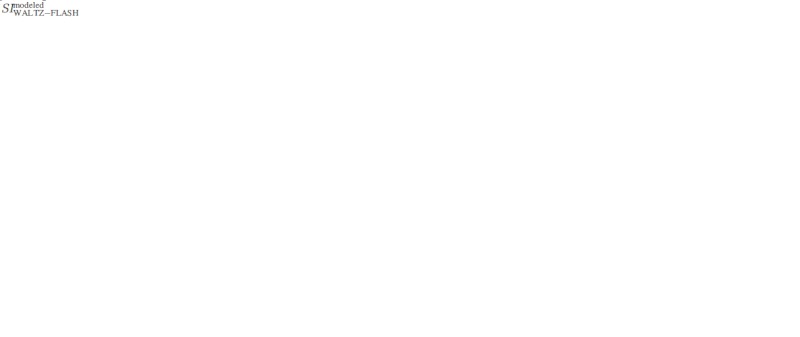
 (*t*), were modeled for the kidney following contrast agent injection. The inputs to the model included the WALTZ-16 pulse parameters (amplitude and duration), the imaging pulse sequence parameters (TR, echo time, and flip angle [α]), the relaxation time constants (*T*_1_, *T*_2_, 

), and the visible PD. Several of these parameters are time dependent as they vary with agent concentration. The agent concentration as a function of time (uptake curve) was defined using data from the mouse kidney, as were the 

 and PD parameters. The *T*_1_ and *T*_2_ relaxation time constants were calculated at each time point using Eqs. 4 and 5 below.

**Table 1:** Relaxation and Exchange Parameters for the Various Proton Pools Used in the Model*

Pool	*T*_2_ (sec)	*T*_1_ (sec)	Chemical shift (ppm)	Exchange rate (Hz)
Bound	0.01	1.0	500	100,000
Amide	0.01	1.0	−50	295
Macromolecular	0.000001	1.0	0	26

* The chemical shift is relative to the free water pool and the exchange rate is the exchange of free water protons with the given pool.

More specifically, the SI 
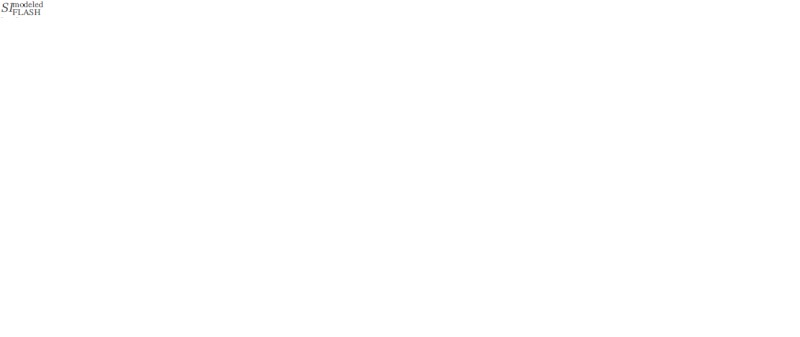
 (*t*) depends on the pulse sequence parameters (TR = 4.71 ms and flip angle α = 11°) and the tissue relaxation parameters (PD, *T*_1_, 

) and was calculated from the FLASH signal Equation (20):


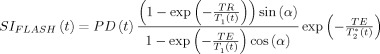
(2)

The SI (
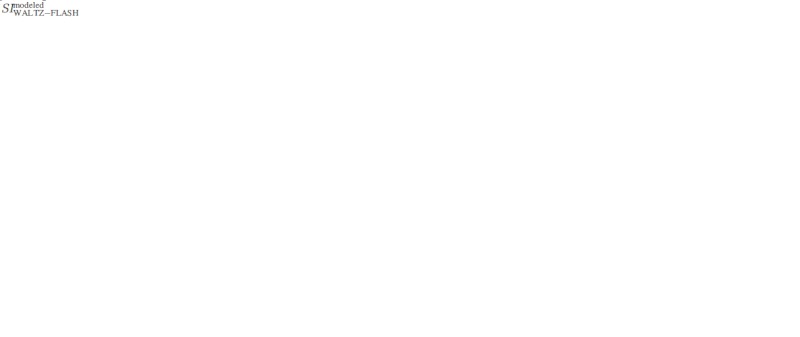
 (*t*)) for the FLASH acquisition preceded by a WALTZ-16 preparation pulse (6-μT amplitude and 240 ms) was also modeled as described above. 
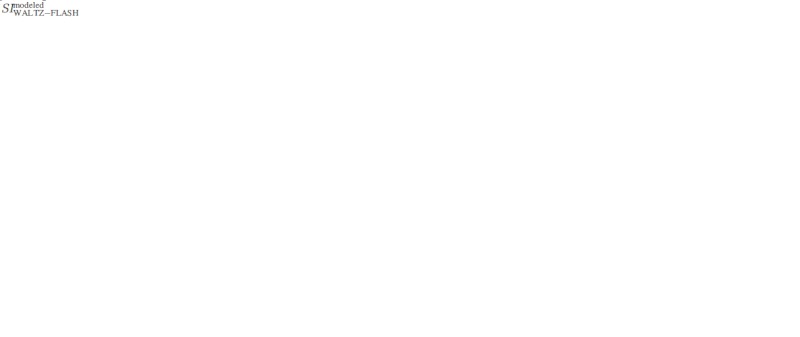
 (*t*)was generated by taking the product of 
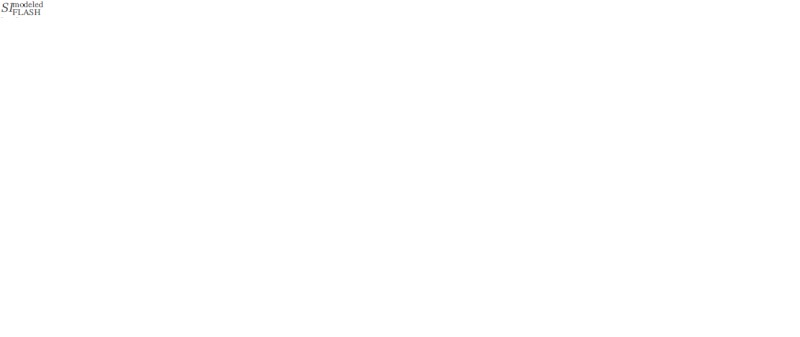
 (*t*)and the fractional signal loss generated by the WALTZ-16 pulse (Exchange(t)), calculated based on the four-pool model described previously (15) and the parameters in Table 1.

The model signal intensities (
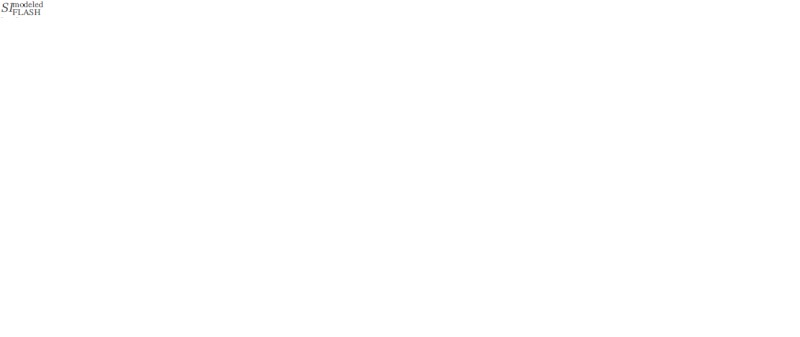
 (*t*)and 
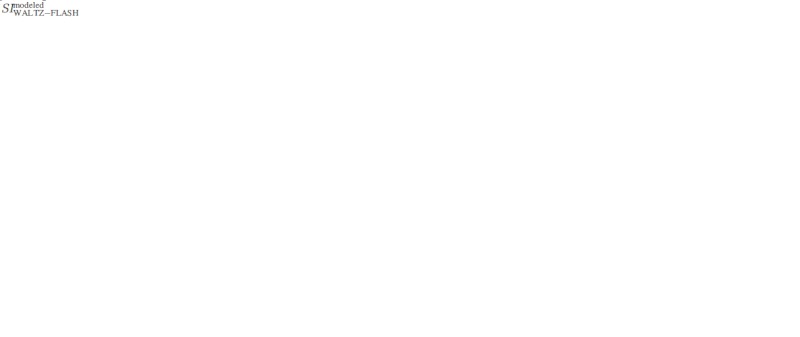
 (*t*)) were compared to the measured SI (*SI*_*FLASH*_ (*t*) and *SI*_*WALTZ*-*FLASH*_ (*t*), respectively) obtained in the kidneys following contrast agent injection. All image analyses and simulations were performed using in-house software written in Matlab (The MathWorks).

## RESULTS

### Phantom Relaxation Measurements

The *T*_1_ and *T*_2_ relaxation time constants quantified in 10% cross-linked bovine serum albumin phantoms were found to vary substantially as a function of agent concentration (Table 2). Equations 3 and 4 give the linear relationship with agent concentration found for both transverse and longitudinal relaxation rates (*R*_1_ = 1/*T*_1_, and *R*_2_ = 1/*T*_2_):


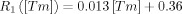
(3)


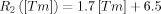
(4)

**Table 2:** *T*_1_ and *T*_2_ Relaxation Time Constants for Tm^3+^-DOTAM-Gly-Lys in 10% Cross-Linked Bovine Serum Albumin

Concentration (mM)	*T*_1_ (sec)	*T*_2_ (sec)
0	2.69	0.16
2	2.67	0.10
6	2.35	0.06
10	2.02	0.004

Equations 5 and 6 give the relationship with agent concentration for *R*
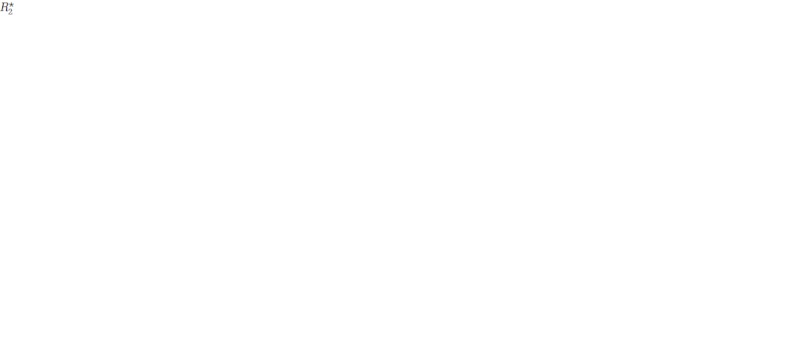
 and PD:


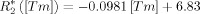
(5)


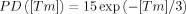
(6)

The OPARACHEE percentage change was calculated and found to be 0, 2.7, 5.1, and 6.7% for the 0−, 2−, 6−, and 10−mM phantoms, respectively. An exponential relation between Tm^3+^ concentration and OPARACHEE percentage change was calculated as:


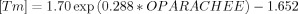
(7)

### Kidney SI Time Course

**FIG. 1:**
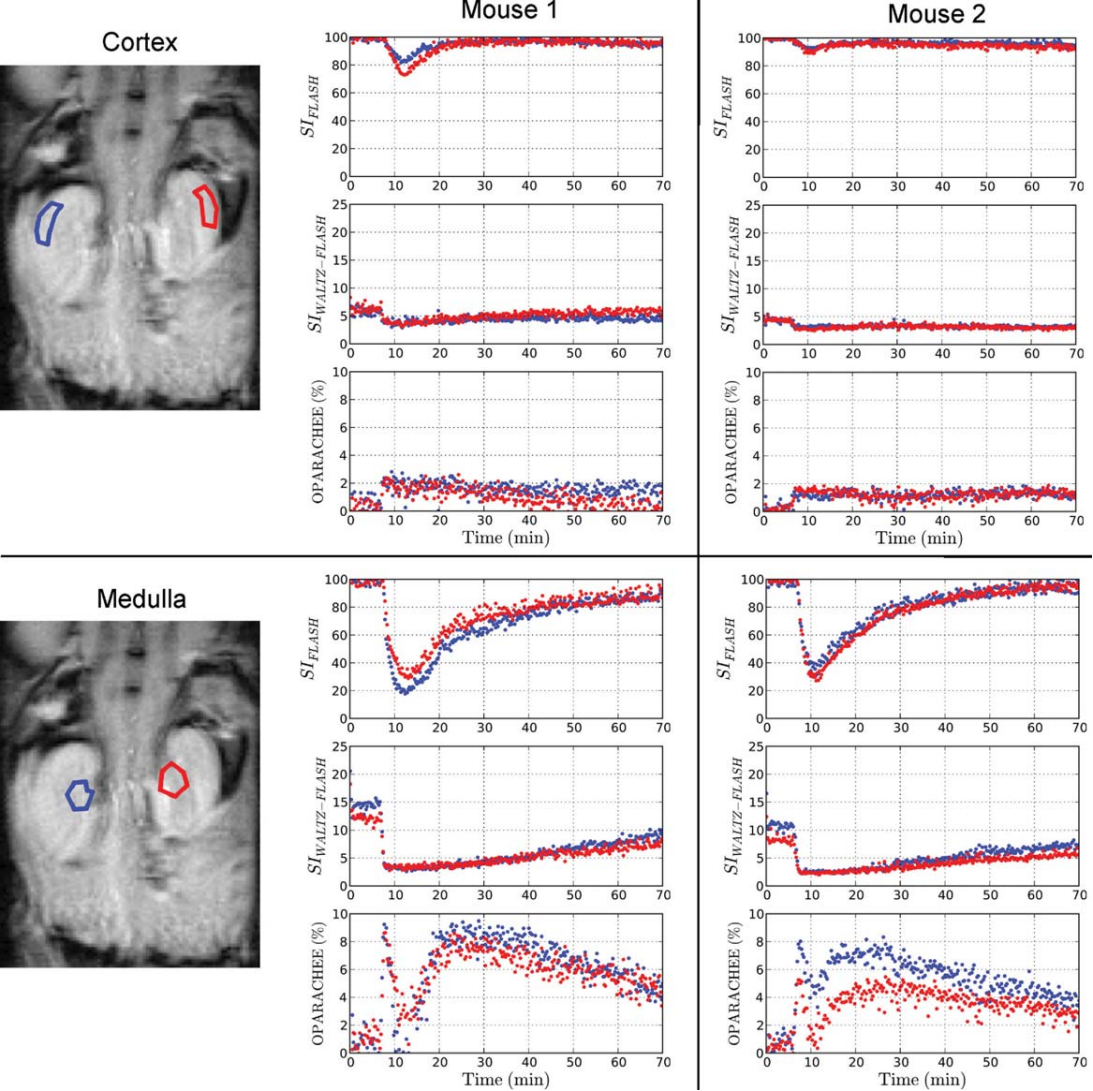
SI time courses shown for the cortex (top row) and medulla (bottom row) regions of the mouse kidney for two different mice. For each set of time courses, there are three plots; the top is the SI measured from the FLASH sequence (no WALTZ-16 pulse) (*SI*_*FLASH*_), the middle is the SI from the FLASH sequence with a WALTZ-16 pulse applied on water resonance (*SI*_*WALTZ*-*FLASH*_), and the bottom is the isolated OPARACHEE effect (i.e., the difference). The data for both the left side (blue) and right side (red) are shown.

Representative in vivo FLASH images and image signal time-courses (*SI*_*FLASH*_ and *SI*_*WALTZ*-*FLASH*_) in the cortex and medulla of the kidney are shown for two animals in Fig. 1. Both *SI*_*FLASH*_ and *SI*_*WALTZ*-*FLASH*_ in the cortex and medulla dropped rapidly approximately 1 min after agent injection (approximately 6 min into the scan session). *SI*_*WALTZ*-*FLASH*_ then increased more slowly than *SI*_*WALTZ*_ to the baseline value. The magnitude of the signal change was far greater in the medulla (25–30% of the baseline value) than the cortex (80–90% of the baseline value). The time courses labeled OPARACHEE in Fig. 1 represent the isolated OPARACHEE signal. In the medulla, the OPARACHEE signal increased rapidly after injection, followed by a brief decrease, after which a more gradual increase and then return toward baseline was observed. In contrast, the cortex had a rapid initial increase followed only by a very gradual decline toward baseline at 70 min. To check the robustness of the correction method used to isolate the OPARACHEE signal, a third animal was scanned during an injection of Gd-DTPA. In this case, the *SI*_*FLASH*_ curve decreased by 10% following injection of the Gd-DTPA complex (data not shown). However, the correction effectively removed this change and produced a residual OPARACHEE effect of <1%, consistent with our expectations.

**FIG. 2:**
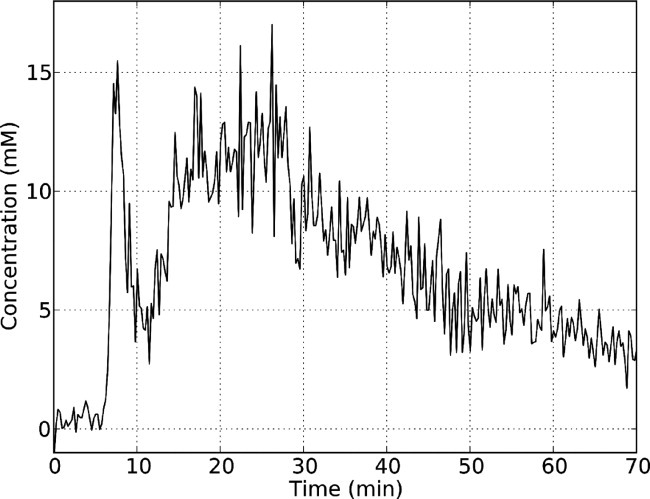
Concentration of Tm^3+^-DOTAM-Gly-Lys agent in the medulla region of the mouse kidney throughout the time course.

The absolute Tm^3+^-DOTAM-Gly-Lys concentration for the left kidney of mouse 2 (Fig. 2) was calculated based on the OPARACHEE effect using the exponential relation defined in Eq. 7.

### Kidney Relaxation Measurement Following Agent Injection

**FIG. 3:**
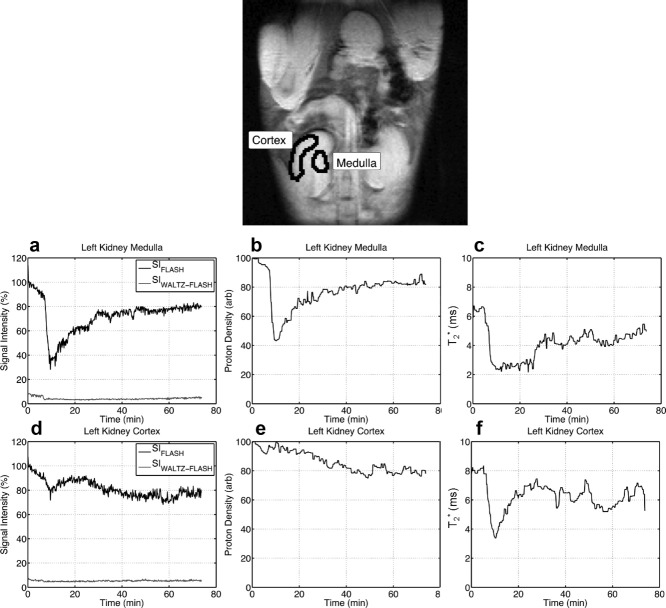
Altered visible PD and 

The in vivo tissue 

 time constant was quantified during agent uptake in the cortex and medulla of the left kidney, as shown in Fig. 3. The image SI associated with the first echo image of the FLASH sequence (*SI*_*FLASH*_) and the FLASH sequence preceded by a WALTZ-16 preparation pulse (*SI*_*WALTZ*-*FLASH*_) is shown as a function of time following agent injection for the left medulla (Fig. 3a) and left cortex (Fig. 3d). The associated visible PD (Fig. 3b and e) and 

 (Fig. 3c and f) vary substantially following contrast agent injection. For the medulla region (top row), the largest signal changes were associated with a decrease in the visible PD, while there were only small changes in the 

. The cortex (bottom row) showed a much smaller decrease in visible PD but a greater and more variable decrease in 

 compared to the medulla.

 following Tm^3+^-DOTAM-Gly-Lys injection. The top image is the first echo of the first image of the time course and is overlaid with the regions of interest used in the plots below. The plots below represent the normalized SI as a function of time (left column), calculated PD (middle column), and calculated 

 (right column) from the left kidney medulla (top row, which corresponds to the medial region of the kidney) and left kidney cortex (bottom row, which corresponds to the distal region of the kidney).

### Model Verification

**FIG. 4:**
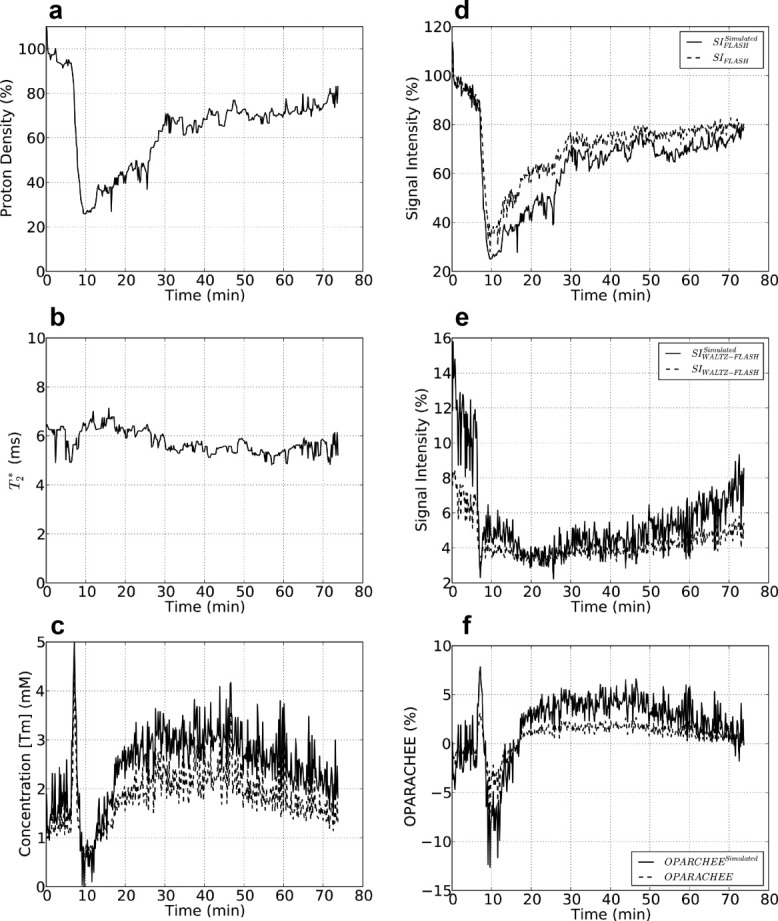
Modeled time-course data for the medulla region of the left mouse kidney. The **(a)** PD, **(b)**

Model input parameters were obtained from the medulla of the left kidney of the fourth animal as a function of time for PD (Fig. 4a) and 

 (Fig. 4b). Additionally, the input concentration curve as a function of time is shown in Fig. 4c. The FLASH SI (*SI*_*FLASH*_., black line in Fig. 4d) and the FLASH SI preceded by the WALTZ-16 pulse (*SI*_*WALTZ*-*FLASH*_, black line in Fig. 4e) were also measured from the same region of the medulla. The OPARACHEE effect was calculated directly from *SI*_*FLASH*_ and *SI*_*WALTZ-FLASH*_ and is shown in Fig. 4f. The simulated signal intensities (
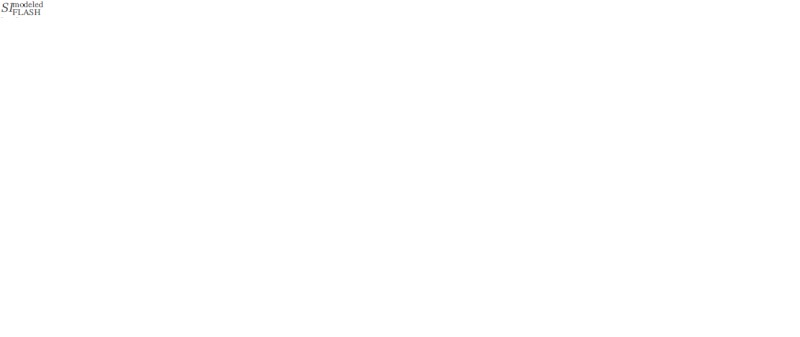
 (*t*) and 
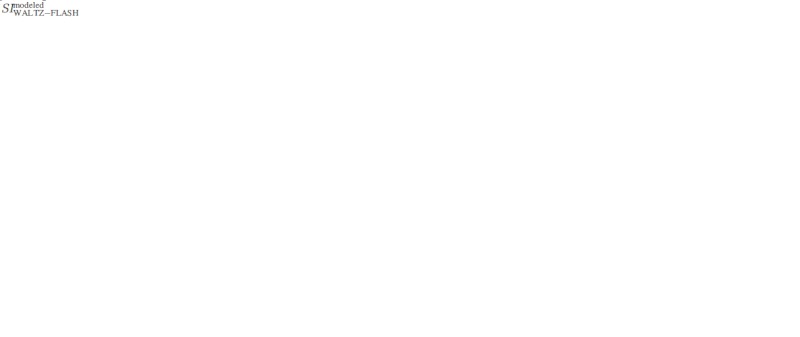
 (*t*)) are represented by the gray curves in Fig. 4d and 4e and showed very good agreement to the measured signal intensities. The OPARACHEE curve was calculated (gray curve in Fig. 4f) from the simulated SI and showed good agreement with the measured OPARACHEE curve (black curve in Fig. 4f). Therefore, the model, which incorporated information from not only the PARACEST effect but also relaxation effects that change as a function of the concentration of the PARACEST agent, accurately recreated the measured SI time courses both with and without the WALTZ-16 preparation pulse.

, and **(c)** uptake curve input functions were calculated from the eight-echo gradient-echo images acquired from mouse 4. The measured (black) and simulated (dashed) signal intensities as shown without WALTZ preparation **(d)** and with WALTZ preparation **(e)**. The measured (black) and simulated (dashed) uptake curves are shown in **(f)**.

## DISCUSSION

The current study demonstrates a significant SI decrease that can be detected in FLASH images acquired at 9.4 T following injection of Tm^3+^-DOTAM-Gly-Lys due to the effect of this compound on bulk water visibility, *T*_1_, *T*_2_, and 

. The inclusion of a WALTZ-16 preparation pulse prior to imaging further decreases SI due to the OPARACHEE mechanism. The OPARACHEE effect can be isolated by calculating OPARACHEE(t) = (*SI*_*FLASH*_(*t*) − *SI*_*WALTZ*-*FLASH*_(*t*))/*SI*_*FLASH*_ (*t*) × 100. The large decrease in image SI (*SI*_*FLASH*_) observed without the use of the WALTZ-16 preparation pulse highlights the need to obtain appropriate reference images to detect OPARACHEE contrast. Such reference images also provide an inherent control for baseline fluctuations of the acquired signal due to drift of the scanner magnetic field over time, which may be beneficial for very long scans. We present here a method for quantifying the OPARACHEE effect independent of changes in tissue relaxation induced by the paramagnetic agent, by alternating between the acquisition of a control scan and a WALTZ-16 prepared scan throughout a time-course study. The difference between subsequent signal intensities (acquired without and with the WALTZ-16 preparation pulse) provides a measure of the OPARACHEE effect independent of relaxation induced signal changes.

### Phantom Relaxation Measurements

The bulk water *T*_1_ and *T*_2_ relaxation time constants were shown to depend linearly on the thulium concentration in 10% bovine serum albumin. The relationship may vary for different tissues and therefore may require separate calibration in each tissue type (e.g., liver, kidney, brain) and different pathologic states (e.g., normal, cancer, stroke). The observed variation in relaxation parameters with agent concentration suggests that care must be taken when attributing signal change in time-course studies directly to the PARACEST or OPARACHEE effect as SI changes will occur even in the absence of a saturation or preparation pulse.

### Kidney SI Time Courses

In the mouse kidney, the variation in image SI following agent injection was dependent on the presence or absence of the WALTZ-16 preparation pulse. With the WALTZ-16 pulse present, *SI*_*WALTZ_FLASH*_ decreased to 55% of the initial value in the cortex and 30% of the initial value in the medulla and then increased linearly over the following 65 min. In the absence of the WALTZ-16 pulse, *SI*_*FLASH*_ had a similar initial decrease but then increased more quickly, returning to the baseline value ∼55 min post injection. Most interesting was the isolated OPARACHEE effect, which showed an initial increase immediately after the injection, then a rapid signal decrease to near baseline around 11 min into the scan, followed by another increase from 12 – 30 min, and finally a gradual decrease over time. The complex nature of the OPARACHEE effect is likely a direct indicator of the concentration of Tm^3+^-DOTAM-Gly-Lys in the kidney as the OPARACHEE effect curve has a similar structure to a renal uptake and excretion time course (21).

### In Vivo Agent Concentration

The in vivo agent concentration was quantified based on an exponential relationship between the OPARACHEE percentage change and agent concentration calculated in phantoms. The relationship was assumed to be exponential over the range of concentrations studied; however, a greater concentration range may require more complex models (e.g., superlinear) to provide an accurate representation of OPARACHEE effect as a function of agent concentration. The OPARACHEE signal calculated from the phantoms was isolated from concomitant changes in bulk water relaxation time constants, and therefore the relationship was expected to be maintained in vivo.

### Visible PD

To further investigate the relaxation mechanisms leading to the decrease in image SI in the absence of the WALTZ-16 preparation pulse, the visible PD and the 

 relaxation time constant were measured following agent injection using a multiecho gradient echo sequence. The visible PD was defined as the y-intercept in the fitted signal decay curve and does not necessarily represent the number of protons in the region. Figure 3b demonstrates that there can be a very large change in the measured intercept as a function of time following agent injection. One explanation for this change is that bulk water protons may become undetectable in the hydration layers surrounding the lanthanide due to an induced shift of the water resonance frequency. A second possible explanation is a dramatic shortening of the *T*_2_ of the hydration layer to the microsecond range such that all signal is dephased prior to the first echo time.

### Relaxation Parameters

The simulations were created based on an input uptake curve and with the relaxation parameters *T*_1_, *T*_2_, and 

, as well as the PD dependent on the agent concentration. The resulting simulated signal intensities with and without WALTZ-16 preparation showed good correspondence to signal intensities measured in mouse kidneys. In addition, fixing any of the relaxation parameters (*T*_1_, *T*_2_, 

, and PD) to a constant value resulted in modeled signal intensities that deviated significantly from the measured signal intensities (data not shown). Therefore, it was necessary to have knowledge of all four parameters for proper modeling of the decay curve.

The isolation of the OPARACHEE effect was achieved by taking the ratio of (*SI*_*FLASH*_(*t*) − *SI*_*WALTZ*-*FLASH*_(*t*))/*SI*_*FLASH*_ (*t*) × 100. Previous studies have incorporated exchange terms into the differential Bloch equations to build three-pool (14) and four-pool (15) models of the PARACEST effect. However, there is currently no analytical solution to this problem. In the Bloch equations, the exchange terms are linearly modeled and therefore the signal change due to the PARACEST effect must be a multiplicative factor applied to the signal equation (Eq. 1). Therefore, the proper method to isolate the OPARACHEE effect (or PARACEST effect) from relaxation effects is to calculate (*SI*_*FLASH*_(*t*) − *SI*_*WALTZ*-*FLASH*_(*t*))/*SI*_*FLASH*_ (*t*) × 100 for each pair of subsequent time points. However, this approach can only be considered an approximation as there is no analytical solution to the modified Bloch equations.

### Limitations

This study focused on a Tm^3+^-based compound (Tm^3+^-DOTAM-Gly-Lys) used to generate OPARACHEE contrast. Image SI was found to change following agent injection, even using a conventional FLASH sequence, due to alterations in bulk water visibility and relaxation time constants induced by the Tm^3+^ lanthanide. Compounds used for detection of the PARACEST effect by off-resonance excitation (e.g., Eu^3+^) were not discussed, but preliminary results indicate a similar, albeit smaller, effect than the effect noted for the Tm^3+^-based compound used in the current study. This result was expected as it depends on the efficiency of a particular compound to alter bulk water visibility and relaxation time constants. The results obtained in the current study are independent of the endogenous macromolecule magnetization transfer (MT) effect and are applicable to all PARACEST agents.

Although the simulations utilized 

 and PD values obtained in vivo, the *T*_1_ and *T*_2_ time constants used were interpolated from the bovine serum albumin data presented in Table 1. It would be optimal to acquire the *T*_1_ and *T*_2_ data in vivo following injection throughout the time course. However, these measurements were not made in the current study due to the length of time required for each.

The current study did not include a correction for amplitude of static field inhomogeneities, which could lead to signal variation within the kidneys (22) and overestimation of the OPARACHEE effect. To minimize such errors, a second-order shim correction was applied to the kidneys, reducing the line width of the bulk water to approximately 150 Hz. In addition, a WALTZ-16 pulse with a bandwidth of ∼400 Hz (2.5-ms subpulse duration) was used for excitation. The WALTZ-16 pulse is inherently insensitive to flip-angle error, and its bandwidth encompassed the range of frequencies found within the kidney, minimizing excitation errors.

### Choice of Imaging Sequence

The current study utilized a FLASH sequence to increase sensitivity to alterations in bulk water *T*_1_ and 

. Pulse sequences that are heavily weighted to relaxation parameters (e.g., FLASH, gradient recalled echo, TFISP) are expected to produce images with large SI variations in the presence of a PARCEST agent, even without the application of a saturation or preparation pulse, and therefore a careful interpretation of signal changes is warranted. The use of fast spin-echo-type sequences can reduce the dependence of image SI on *T*_1_ and *T*_2_ relaxation time constants; however, such sequences will not protect against signal loss due to the decreased visibility of the bulk water pool in the presence of a lanthanide-based agent. Therefore, all such experiments should include careful controls to account for such changes.

### Increasing Sensitivity of Detection

The SI change was shown to be a function of relaxation parameters, and therefore image SI will depend on the type of pulse sequence used for acquisition. The FLASH pulse sequence used in the current study was very sensitive to *T*_1_ relaxation. However, there are many other possibilities for image acquisition, including TFISP and spin-echo-based sequences. An on-resonance spin-echo pulse sequence with a long TR would have the smallest dependence on relaxation parameters and consequently the cleanest detection of OPARACHEE contrast. However, the acquisition time of such sequences would be relatively long (16). On the other hand, short TR pulse sequences such as FLASH and TFISP would be more sensitive to relaxation parameters but would be more rapid. The timing parameters of such sequences could potentially be modified to provide optimal in vivo detection sensitivity of the OPARACHEE or PARACEST effect. Such optimization would require knowledge of the relaxation properties of a particular agent. The SI dependence on pulse sequence acquisition and agent properties will be addressed in future work.

## CONCLUSIONS

SI time-courses without WALTZ-16 preparation showed large SI changes as a function of time following PARACEST contrast agent injection. This signal variation was attributed to changes in relaxation and bulk water visibility as the agent concentration varied. Despite these effects, on-resonance PARACEST agent detection (OPARACHEE contrast) can be isolated by the acquisition of control images throughout the time course to account for agent-induced signal changes from altered bulk water visibility, *T*_1_, *T*_2_, and 

 relaxation mechanisms. Failure to account for these signal changes can lead to the significant overestimation of the PARACEST effect. The relationship between OPARACHEE contrast and agent concentration, independent of relaxation parameters, was used to quantify absolute agent concentration changes throughout a time course in a mouse kidney.
